# Antioxidant Properties of Embelin in Cell Culture. Electrochemistry and Theoretical Mechanism of Scavenging. Potential Scavenging of Superoxide Radical through the Cell Membrane

**DOI:** 10.3390/antiox9050382

**Published:** 2020-05-05

**Authors:** Francesco Caruso, Miriam Rossi, Sarjit Kaur, Emmanuel Garcia-Villar, Nora Molasky, Stuart Belli, Joanna D. Sitek, Fabio Gionfra, Jens Z. Pedersen, Sandra Incerpi

**Affiliations:** 1 Vassar College, Department of Chemistry, Poughkeepsie, NY 12604, USA; rossi@vassar.edu (M.R.); sakaur@Vassar.edu (S.K.); egarciavillar@vassar.edu (E.G.-V.); nmolasky@uw.edu (N.M.); belli@vassar.edu (S.B.); 2 Department of Renal and Body Fluid, Mossakowski Medical Research Centre, Polish Academy of Sciences Physiology, 02-106 Warsaw, Poland; jsitek@imdik.pan.pl; 3 Department of Sciences, University Roma Tre, 00146 Rome, Italy; fabio.gionfra@uniroma3.it (F.G.); sandra.incerpi@uniroma3.it (S.I.); 4 Department of Biology, University Tor Vergata, 00133 Rome, Italy; jenszacho.pedersen@uniroma2.eu

**Keywords:** embelin, antioxidant, cyclic voltammetry, reactive oxygen species, cytoprotection, THP-1 monocytes, BV-2 murine microglia, DFT, *Embelia ribes*, superoxide

## Abstract

Embelin, a plant natural product found in *Lysimachia punctata* (Primulaceae), and *Embelia ribes* Burm (Myrsinaceae) fruit, possesses interesting biological and pharmacological properties. It is a unique chemical species as it includes both quinone and hydroquinone functional groups plus a long hydrophobic tail. By using hydrodynamic voltammetry, which generates the superoxide radical in situ, we show an unusual scavenging capability by embelin. Embelin as a scavenger of superoxide is stronger than the common food additive antioxidant 2,6-bis(1,1-dimethylethyl)-4-20 methylphenol, (butylated hydroxytoluene, BHT). In fact, embelin is even able to completely abolish the superoxide radical in the voltaic cell. Computational results indicate that two different types of embelin scavenging actions may be involved, initially through π–π interaction and followed by proton capture in the cell. A related mechanism describes embelin’s ability to circumvent superoxide leaking by transforming the anion radical into molecular oxygen. In order to confirm its antioxidant properties, its biological activity was tested in a study carried out in THP-1 human leukemic monocytes and BV-2 mice microglia. A 3-(4,5-dimethylthiazol-2-yl)-2,5-diphenyltetrazolium bromide (MTT) assay, proliferation curves and antioxidant activity by the use of a fluorescent probe showed good antioxidant properties at 24 h. This suggests that embelin’s long alkyl C10 tail may be useful for cell membrane insertion which stimulates the antioxidant defense system, and cytoprotection in microglia. In conclusion, embelin could be an interesting pharmacological tool able to decrease the damage associated with metabolic and neurodegenerative diseases.

## 1. Introduction 

**Scheme 1:**
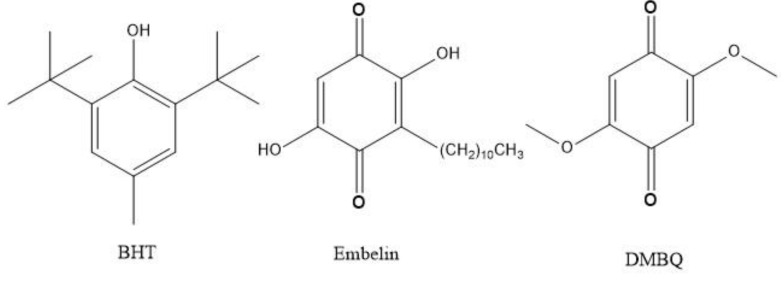
Compounds analyzed in this work.

Embelin (2,5-dihydroxy-3-undecyl-*p*-benzoquinone) is a bioactive molecule from several plant sources including *Lysimachia punctata* (Primulaceae) and *Embelia ribes* Burm from Myrsinaceae. The chemical structure of embelin (Scheme 1) consists of a polar dihydroxy-1,4-benzoquinone ring, that is, two carbonyl oxygen atoms, as adjacent to the two vinyl hydroxyl groups. Therefore, it is a *p*-quinone and also a sort of hydroquinone, which makes this compound unique as a radical scavenger. Embelin also possesses a non-polar long alkyl saturated chain which confers an important lipid character potentially useful for membrane insertion. 

Embelin possesses a wide spectrum of medicinal and pharmacological features including anti-inflammatory, analgesic, antitumor in in vivo and in vitro models of carcinogenesis, antioxidant, anxiolytic, antibacterial, anticonvulsant and antidepressant properties [1,2]. As an example, treating glioma cells with either (25 μM) embelin, tumor necrosis factor-related apoptosis-inducing ligand (TRAIL) or the combination of both, primed the malignant glioma cells to TRAIL-mediated apoptosis [3,4]. Some studies have shown that embelin has prooxidative effects on certain types of cells [5,6]. Furthermore, studies have shown anti-diabetic activity and demonstrated a good correlation between oxidative stress and the development of type 2 diabetes and its late complications [7]. 

In general, when studying radical scavenging properties, a hydroxyl group is considered useful for its release of a H atom, making the scavenged radical less reactive. Additionally, if an ortho or para H-atom is available for abstraction, as in the case of the 3,4-dihydroxyphenyl moiety of quercetin, it can yield a quinone structure. In contrast, as described recently through structural and computational analyses [8], embelin is not able to react in this classical way with superoxide. Embelin scavenges the superoxide radical in an unusual manner: by abstracting its electron and releasing molecular oxygen. One reason for this behavior comes from the fact that both embelin hydroxyl groups establish strong intramolecular H-bonds with the carbonyl neighbors, and so become less available for scavenging. Interestingly, the defensive power of embelin against paraquat-incited lung damage was studied in relationship with its antioxidant and anti-inflammatory action. Oxidative stress markers, like malondialdehyde (MDA), antioxidative enzymes, such as superoxide dismutase (SOD), catalase (CAT), and glutathione peroxidase (GSH Px), inflammatory cytokines, for example interleukin-1β (IL-1β), tumor necrosis factor-α, and IL-6, along with histological examination, and nuclear factor kappa B/mitogen-activated protein kinase (NF-κB/MAPK) gene expression were evaluated in lung tissue [9,10]. Embelin treatment significantly decreased MDA and increased SOD, CAT, and GSH Px. Embelin appreciably reduced levels of inflammatory cytokines in paraquat-administered and paraquat-intoxicated rats. The outcomes show the impact of embelin inhibitory action caused by paraquat, a well-known inducer of superoxide radical. 

Embelin has significant molecular flexibility as seen by the difference in torsion angle between the ring and the alkyl chain of 67° in the molecular crystal [8], which differs markedly from when it is embedded in the plasminogen activator inhibitor-1 (PAI-1) binding site (almost planar—with about a 10° torsion angle) [11]. Such molecular flexibility by embelin gives support for building a membrane model below, in which we demonstrate embelin’s potential to alleviate the aging condition in the mitochondria. 

Neurodegenerative disorders such as Parkinson’s, Alzheimer’s, and Huntington’s disease have altered mitochondrial function, essential for the ATP production by the cells, particularly in nervous cells that have intense firing activity, reactive oxygen species (ROS) production and calcium level regulation, important for the release of neurotransmitters in the synaptic cleft. Mitochondrial dysfunction with decrease of ATP production and oxidative stress is involved in these pathologies. Functional peroxisomes proliferator–activator gamma receptor coactivator -1 α (PGC-1α) is essential for all cells and is a master regulator of mitochondrial function. Embelin has been reported also to be a mild uncoupler and, therefore, a stimulator of mitochondrial biogenesis [2,12]. Embelin shows some structural analogy to coenzyme Q10. This entire body of evidence prompted us to study the possible protective effects of this compound in THP-1 human leukemic monocytes and BV-2 murine microglia that represent the immune system of the central nervous system [12].

Our data show that embelin is not toxic at the concentration employed in microglia cells and has a protective effect toward oxidative insult by reacting with the superoxide. 

## 2. Materials and Methods

### 2.1. Chemicals

Embelin, (Indofine Chemicals, NJ, USA). Dimethyl sulfoxide (DMSO, anhydrous, ≥99.9%), tert-butyl ammonium bromide (TBMA), [(2,2-dimethyl-6,6,7,7,8,8,8-heptafluoro-3,5-octanedionato)silver(I)] (Sigma-Aldrich, St. Louis, MO, USA). Roswell Park Memorial Institute medium (RPMI 1640), Dulbecco’s modified Eagle medium (DMEM), streptomycin (100 mg/mL), penicillin (100 U/mL), D-glucose, cumene hydroperoxide, 3-(4,5-dimethylthiazol-2-yl)-2,5-diphenyltetrazolium bromide (MTT), phosphate buffered saline (PBS)- one tablet/L buffer without Calcium and Magnesium (Sigma-Aldrich, St. Louis, MO, USA). 2’,7’-dichlorodihydrofluorescein diacetate (DCFH2-DA) (Molecular Probes, Eugene, OR, USA). Sterile plasticware for cell culture (Falcon Brand, San Diego, CA, USA); fetal bovine serum (GIBCO, Grand Island, NY, USA).

### 2.2. Equipment

Hydrodynamic voltammetry at a rotating ring-disk electrode (RRDE) was carried out using the Pine Research (Pine Research, Durham, NC, USA) WaveDriver 20 bipotentiostat with the Modulated Speed Electrode Rotator. The working electrode is the AFE6R2 gold disk and gold ring rotator tip (Pine Research, Durham, NC, USA), combined with a coiled platinum wire counter electrode and a reference electrode consisting of an AgCl coated silver wire immersed in 0.1 M TBAB in dry DMSO in a fritted glass tube. The electrodes were placed in a 5-neck electrochemical cell together with means for either bubbling or blanketing the solution with gas. Voltammograms were collected using Aftermath software provided by Pine Research. Careful cleaning of the electrodes was performed by polishing with 0.05 µm alumina-particle suspension, Allied High Tech Products, Inc. (Rancho Dominguez, CA, USA), on a moistened polishing microcloth to eliminate potential film formation [13].

### 2.3. RRDE Study

Stock solutions of embelin 0.1 M in anhydrous DMSO were used in trials. For the experiment, a solution of 0.1 M TBAB in anhydrous DMSO is bubbled for 5 min with a dry O_2_/N_2_ (35%/65%) gas mixture to establish the dissolved oxygen level in the electrochemical cell. The Au disk electrode was then rotated at 1000 rpm while the disk was swept from 0.2V to −1.2Volts and the ring was held constant at 0 Volts, the disk voltage sweep rate was set to 25mV/s. The molecular oxygen reduction peak (O_2_ + e^−^ → O_2_^−•^) was observed at the disk electrode at −0.6 volts; the oxidation current (O_2_^−•^ → O_2_ + e^−^) is observed at the ring electrode. An initial blank is run on this solution and the ratio of the peak ring current to disk current is calculated as the “collection efficiency” in the absence of antioxidant. Next, an aliquot of the antioxidant is added, the solution is bubbled with the gas mixture for 5 min and the cyclovoltammogram is rerecorded. Again, the reduction and oxidation peaks are measured and the collection efficiency is calculated. Any decrease in the collection efficiency is due to the amount of superoxide removed by the antioxidant. 

### 2.4. Computational Study

Calculations were performed using programs from Biovia (San Diego, CA, USA). Density functional theory (DFT) code DMol^3^ was applied to calculate energy, geometry, and frequencies implemented in Materials Studio 7.0 [14]. We employed the double numerical polarized (DNP) basis set that included all the occupied atomic orbitals plus a second set of valence atomic orbitals, and polarized d-valence orbitals [15]; the correlation generalized gradient approximation (GGA) was applied including Becke exchange [16] plus Perdew correlation [17] (PBE). All electrons were treated explicitly and the real space cutoff of 5 Å was imposed for numerical integration of the Hamiltonian matrix elements. The self-consistent field convergence criterion was set to the root mean square change in the electronic density to be less than 10^−6^ electron/Å^3^. The convergence criteria applied during geometry optimization were 2.72 × 10^−4^ eV for energy and 0.054 eV/Å for force. All calculations included the effect of DMSO as solvent to allow for the correlation with the experimental features from cyclovoltammetry results.

### 2.5. Cells in Culture

BV-2 murine microglia cells were a kind gift from Dr. Tiziana Persichini, Department of Sciences, University Roma Tre, Rome, Italy. The cells were maintained in the logarithmic phase of growth in Dulbecco’s modified Eagles’s Medium (DMEM) containing 4.5 g/L glucose, supplemented with 10% FBS and 100 μg/mL streptomycin and 100 U/mL penicillin, in a humidified atmosphere of 5% CO_2_ at 37 °C. Confluent cultures were passaged twice a week by mild trypsinization and re-seeded in T25 culture flasks, according to the product sheet of ATCC. 

L6 myoblasts from rat skeletal muscle and human leukemic monocytes THP-1 were obtained from the American Type Culture Collection (ATCC, Rockville, MD, USA). L6 myoblasts were seeded in 75 cm^2^ tissue culture flasks and grown in Dulbecco’s modified Eagle’s medium (DMEM) containing 4.5 g/L glucose, supplemented with 10% heat-inactivated fetal bovine serum, 100 µg/mL streptomycin, and 100 U/mL penicillin, in an atmosphere of 5% CO_2_ at 37 °C. Cells reached confluency after 5 days (approximately 6.0 × 10^6^ cells/flask) and were kept in culture as myoblasts by continuous passages at preconfluent stages as previously reported [18,19].

Human leukemic monocytes, THP-1, were grown in suspension in RPMI-1640 medium with 10% FBS, 100 µg /mL streptomycin and 100 U/mL penicillin, in a humidified atmosphere with 5% CO_2_ at 37 °C [18,19]. THP-1 monocytes were passaged twice a week by 1:4 dilutions and re-seeded; cells from passages 7-23 were used for the experiments.

### 2.6. Intracellular ROS Determination 

Reactive oxygen species (ROS) were measured both in BV-2 and THP-1 cells. The method used was a standard assay based on the intracellular fluorescent probe DCF [18,19]. For BV-2 cells, the medium was discarded and cells were washed twice with 5 mL phosphate buffered saline (PBS) containing 5.0 mM glucose (PBS-glucose), at 37 °C. Cells were gently scraped off with PBS-glucose at 37 °C and centrifuged at 1200 rpm for 5 min, the supernatant was discarded and the pellet resuspended in PBS-glucose. The THP-1 monocytes were centrifuged at 1200 rpm for 10 min; the supernatant was discarded and cells were washed twice with 5 mL PBS-glucose at 37 °C to remove the serum that could affect the action of the fluorescent probe. After the last centrifugation, the pellet was resuspended in PBS-glucose and the probe DCFH2-DA was added at 10 µM final concentration (from a stock solution of 10 mM in dimethyl sulfoxide). The incubation was carried out for 30 min in the dark at 37 °C. From that point onwards the protocols for the two cell types were identical. At the end of the incubation cells were washed twice, centrifuged at 1200 rpm for 5 min, and the final cell pellet was resuspended in PBS-glucose. Before the experiments cells recovered at room temperature for 1 h. ROS production was measured in a Perkin-Elmer Multilabel Counter Victor3V Wallac 1420, by evaluating the intracellular DCF fluorescence. We measured the ability of embelin to buffer the ROS production in cells exposed to the radical generator cumene hydroperoxide (200 µM) at different times (10 min, 30 min, 1 h, 3 h and 24 h). Before addition of cumene hydroperoxide, cells were pre-incubated in the cuvette with embelin at 37 °C for 10 min, as reported earlier for other compounds [18].

The antioxidant activity of embelin was determined by the decrease of intracellular DCF fluorescence in the different experimental conditions. Embelin did not affect the fluorescence on their own. In a separate series of experiments, we ensured that DMSO concentrations had no effect on evoked responses.

### 2.7. Proliferation Studies

BV-2 microglia cells were seeded in 60 × 15 mm Petri dishes (1.0–1.5 × 10^5^ cells/dish) and grown in medium supplemented as reported above. The following day the medium was discarded, and 1 mL of new medium containing different concentrations of embelin was added. The cells were counted every 24 h, in a Neubauer chamber, after mild trypsinization. THP-1 cells were seeded in a 24 multiwell (6.0–6.5 ×10^4^ cells/well) and grown in medium supplemented as reported above. THP-1 monocytes were stimulated with embelin the day after seeding and counted up to 96 h from seeding, using a Neubauer chamber, after a mild resuspension.

### 2.8. MTT Assay

Cell viability and the possible cytotoxic effect of embelin were assessed by the MTT assay [19,20]. L-6 cells were seeded in 96-wells plates at 10,000 cells/well in 200 µL DMEM containing 10% serum. The day after seeding the medium was discarded and 100 µL of new medium containing cumene (27.5 µM) and different concentrations of embelin were added to each well, depending on the specific experimental condition as reported in a previous paragraph. Then MTT solution (5 mg/mL in PBS) was added at the final concentration of 10% with respect to the total volume, and incubation was carried out at 37 °C for 3–4 h in the dark. During the incubation, there was a conversion of the yellow MTT to purple formazan by the mitochondrial succinate dehydrogenase of living cells. Then lysis buffer (DMSO containing ammonia) [20] was added and further incubation at 37 °C for 30 min in the dark was carried out. Cells were then resuspended and the optical density was read with an ELISA-reader at 550–570 nm. Results are reported as the mean ± SD of 2 experiments carried out in quintuplicate, unless otherwise specified.

### 2.9. Statistical Analysis

The results of the experiments with the cells are reported as mean ± SD and analyzed by one-way analysis of variance (ANOVA), followed by post-hoc Bonferroni’s Multiple Comparison Test or by the Student’s t-test; the analyses were carried out using the Prism4 statistics program. Differences were considered significant at *p* < 0.05.

## 3. Results

### 3.1. Electrochemistry

The antioxidant capability of embelin towards the superoxide radical was studied following a recently developed method, that uses a variation of cyclovoltammetry called Rotating Ring Disk Electrode (RRDE) [21]. Essentially, the superoxide radical is generated in a voltaic cell using anhydrous DMSO as solvent, and bubbling a controlled amount of oxygen. The superoxide radical is obtained at enough negative potential so that O_2_ captures an electron from the working electrode

O_2_ + e^−^ → O_2_^•−^ (1)

The configuration of the voltaic cell includes, besides the working rotating disk electrode, a reference electrode and a ring electrode around the disk. The ring is set so that the reaction opposite to **(1)** is performed, that is the potential is chosen positive enough for oxidation of the superoxide. The progressive amounts of antioxidant added, in this case embelin, to the voltaic cell account for the consumed superoxide (at the ring electrode), which is detected more precisely than when using only one working electrode in classical cyclovoltammetry [21]. 

One of the four compounds analyzed, butylated hydroxytoluene, BHT, Scheme, is used as a food additive and also to prevent oxidation in fluids such as fuel. The active scavenger moiety of BHT is its hydroxyl group, which is able to release its H atom to superoxide, as seen below.

**Figure 1:**
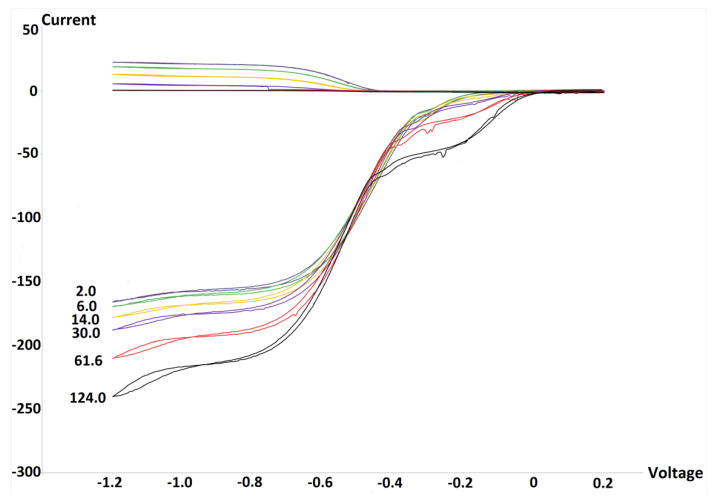
Rotating ring-disk electrode (RRDE) graph of embelin. Each run has a specific color, for instance, the yellow color is associated with 14.0 × 10^−5^ M concentration, and is consistently shown above, at the ring electrode. The bulge centered at −0.2 volts is probably due to capture of an electron by embelin, as demonstrated recently by the quinone emodin [22], and so the whole effect is due to sequestering superoxide plus embelin reduction.

Embelin is a benzoquinone, Scheme 1, and contains two vinyl hydroxyls. We previously have shown that embelin hydroxyls are not able to release any H atom, rather it is through a π–π interaction that superoxide releases an electron to the quinone ring while forming O_2_ [8]. From the RRDE graph, Figure 1, it is seen that progressive amounts of embelin decrease and even deplete the superoxide content of the voltaic cell. That is, the signal detected at the ring electrode indicates that the superoxide is not consumed by the antioxidant. This is located at the upper part of the graph, and shows that at 124.0 × 10^−5^ M of embelin, the superoxide is completely eliminated.

To further clarify the mode of action of embelin, a derivative was studied where both hydroxyls were methoxylated and the long alkyl chain is suppressed. This embelin derivative is 2,5-dimethoxybenzoquinone, DMBQ, Scheme 1, and it was also studied using the RRDE method. Since no hydroxyls are in DMBQ, we expect that its scavenging of superoxide will be similar to that of embelin, that is, through electron transfer of superoxide to the quinone ring.

**Figure 2:**
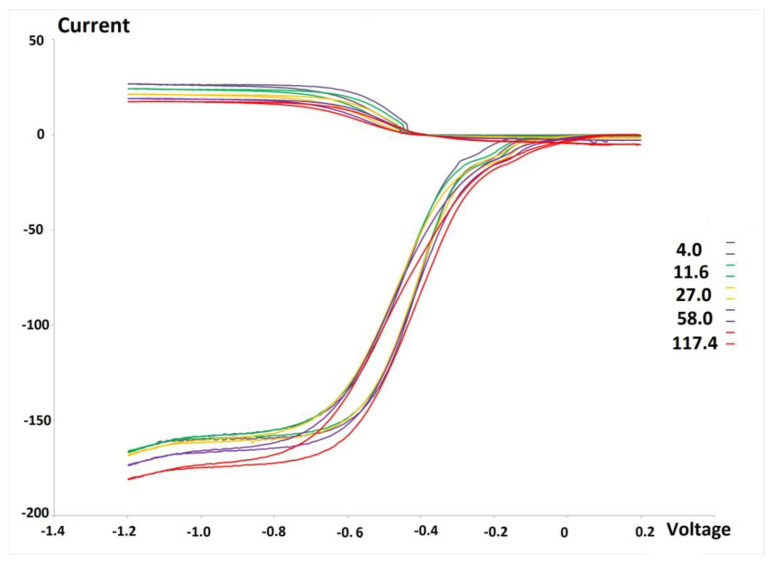
RRDE graph of 2,5-dimethoxybenzoquinone (DMBQ). Colors are associated with (10^−5^) specific molarity, for instance, the maximum concentration is 117.4 × 10^−5^ M (red color), and the whole range is 4.0—117.4 × 10^−5^ M. Voltage measured in volts, Currency in μA.

Figure 2 shows the voltammogram results for DMBQ. At the maximum concentration of 117.4 × 10^−5^ M there is still a marked amount of superoxide in the voltaic cell, which indicated DMBQ is a weaker scavenger than embelin for superoxide.

An additional experiment used toluene as potential scavenger of superoxide, and there was no decrease of superoxide with toluene aliquots, which means toluene is not a scavenger, as seen in Figure S1. In fact, toluene scavenging is null, even at the highest concentration among the four studied species, 405.0 × 10^−5^ M.

**Figure 3:**
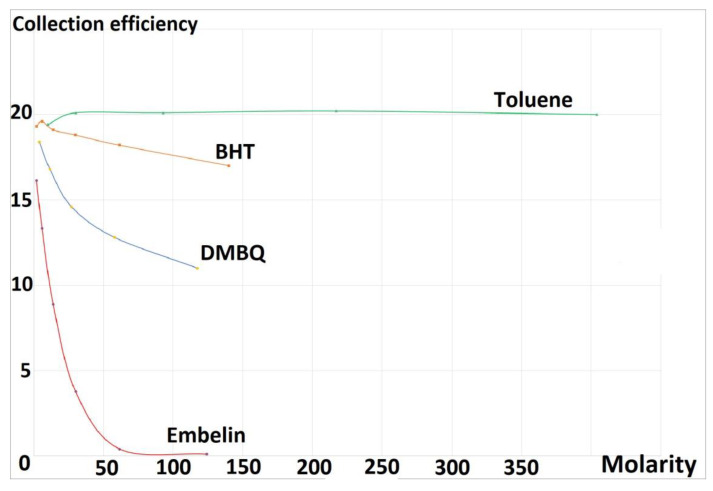
Collection efficiency of the four analyzed species. Molarity expressed as 10^−5^ times.

A summary of RRDE experiments is shown in Figure 3. This is the collection efficiency, obtained by dividing current values at the disk and at the ring for each antioxidant aliquot added, which means for a given concentration of the scavenger, there is a datum at the graph. The best descriptor of the antioxidant capability in the graph is the slope of the curve, which is generally linear for flavonoids [21]. Although the protocol followed in all four experiments was identical, the initial amount of superoxide detected is not coincident, Figure 3, and so the collection efficiency origin (zero molarity, corresponding to no antioxidant added to the voltaic cell) is different for the four species. This is in part due to ambient humidity, which is very strong in the Hudson Valley during the whole year, as water destroys superoxide. However, the linear slope, always found at low concentration, is not affected by a potential loss of superoxide in the voltaic cell due to moisture or other factors. The slopes of Figure 3 are presented in Table 1. For reference, the quercetin RRDE experiment was also performed, using the same protocol, resulting in −15.4 × 10^4^, which is higher than for embelin. We note that toluene and BHT have linear behavior, while embelin and DMBQ do not, at a higher concentration. 

### 3.2. Density Functional Theory

**Figure 4:**
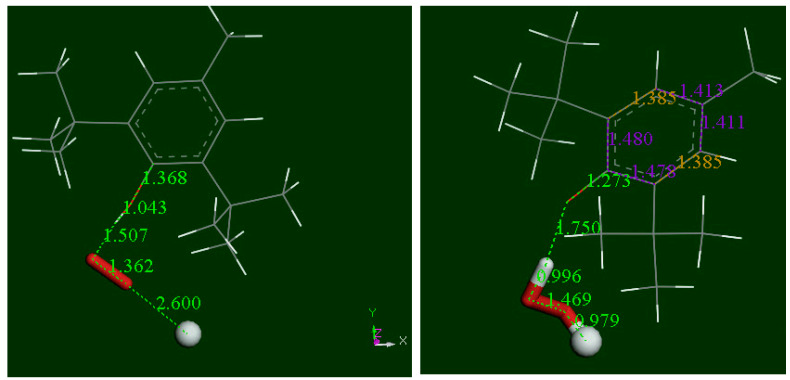
DFT geometry pathway of BHT scavenging the superoxide radical. *Left:* Minimum obtained after geometry optimization from posing superoxide (stick style, original d(O-O) =1.373 Å becomes 1.362 Å) to BHT H(hydroxyl). A proton (ball style) has been added ready for further reaction, with reacting atoms separated 2.60 Å. *Right,* the minimum reached after geometry optimization of left system showing formation of H_2_O_2_, and having no energy barrier. The scavenger shows a short C-O bond length 1.273 Å, compared with single C-C bond, 1.362 Å, at the reactant. This sort of carbonyl bond influences the ring structure with C-C bonds, 1.478 Å 1.480 Å (adjacent) and farther away both = 1.411 Å (purple color); they are longer than the two intermediate bonds (both 1.385 Å, yellow color).

Our RRDE analysis includes BHT scavenging, which is weaker than for embelin. BHT is a widely used antioxidant [23] and is expected to scavenge superoxide in the classical manner by releasing its H(hydroxyl) atom to become a radical, with the unpaired electron localized within the aromatic ring, and meanwhile forming HOO^−^. Figure 4 (left), shows the minimum geometry grouping obtained after geometry minimization for a BHT attack by superoxide. At this point, a proton was included in the system, as shown in Figure 4 (left), to study a potential reaction evolution through geometry optimization. This result is shown in Figure 4 (right), which depicts the formation of H_2_O_2_ and the BHT radical; this process has no energy barrier. 

**Figure 5:**
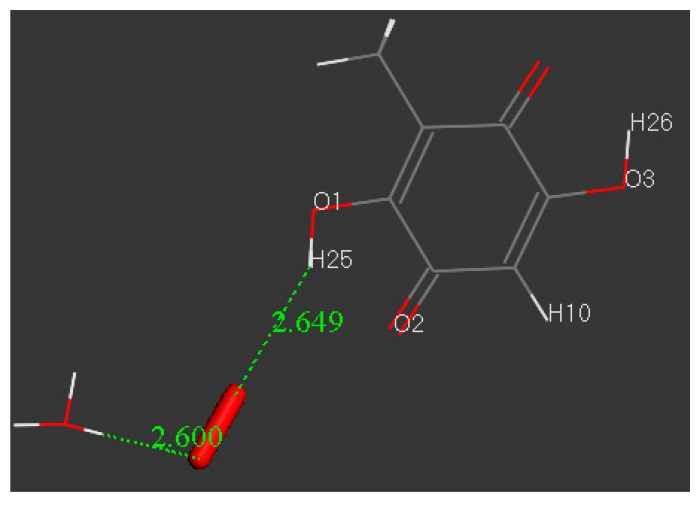
An attempt to force, on an embelin model, the σ scavenging of superoxide by adding a hydronium species, initially located at van der Waals separation of 2.60 Å.

As already described [8], embelin hydroxyls are not suitable for scavenging superoxide as the initial separation between H(embelin) and O(superoxide), 2.60 Å, becomes 2.649 Å, which means the radical is rejected. We further examined this feature by approaching a hydronium species to the rejected configuration, shown in Figure 5. To simplify this calculation, we used a model of embelin with the C_10_ alkyl chain replaced by a methyl group.

**Figure 6:**
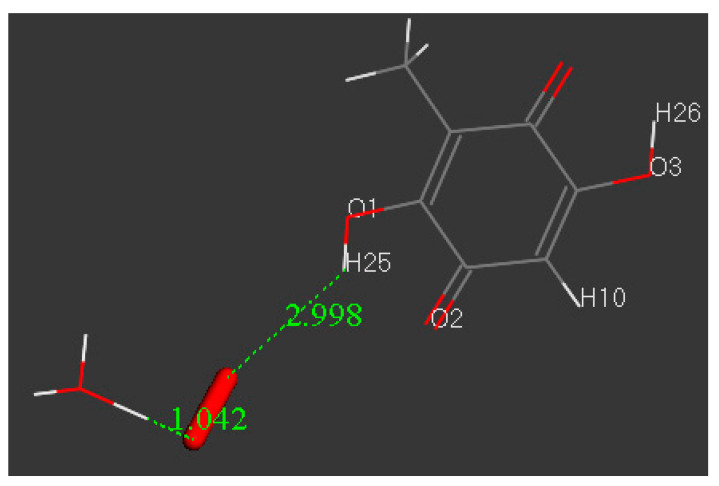
After the system shown in Figure 5 is minimized, a H(hydronium) is captured and a O_2_H species (radical or cation) is formed, which drifts further apart from embelin, 2.998 Å.

After H_3_O^+^ was posed to O(superoxide), the system was geometry-optimized and the results were a minimum of energy, as shown in Figure 6. This further confirms that, at least initially, embelin hydroxyls have no role in scavenging superoxide, as already described [8].

**Figure 7:**
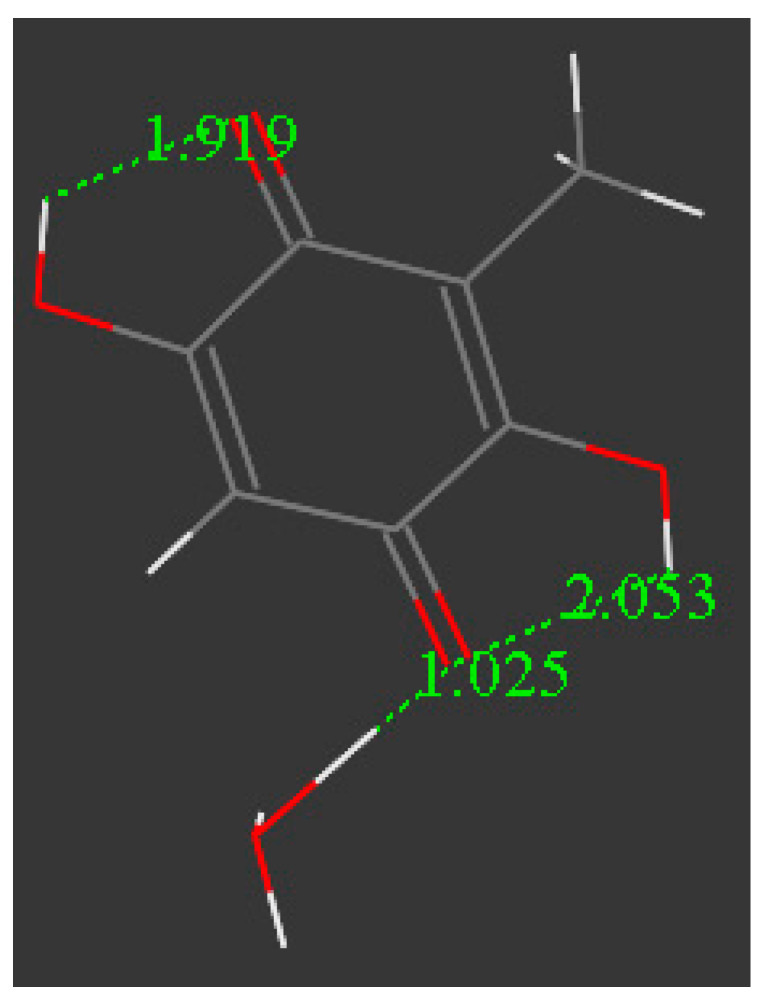
After superoxide donates the electron to the embelin-model ring, H_3_O^+^ is reacted at one O(carbonyl), and the proton is captured. The formed species, [embelin-model-H]^•^, is a radical of neutral charge and this reaction has a ΔG of −31.4 kcal/mol and no energy barrier,.

**Figure 8:**
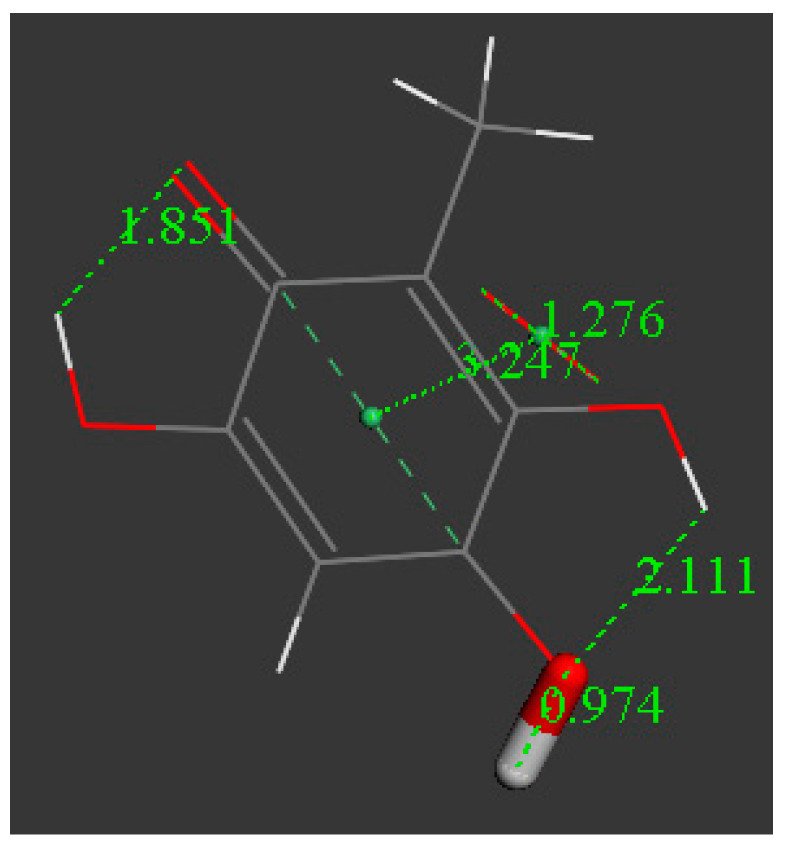
A second superoxide is also able to donate an electron to the ring, and the formed species is a radical of charge −1, [embelin-model-H]^−^ ΔG = −38.1 kcal/mol.

**Figure 9:**
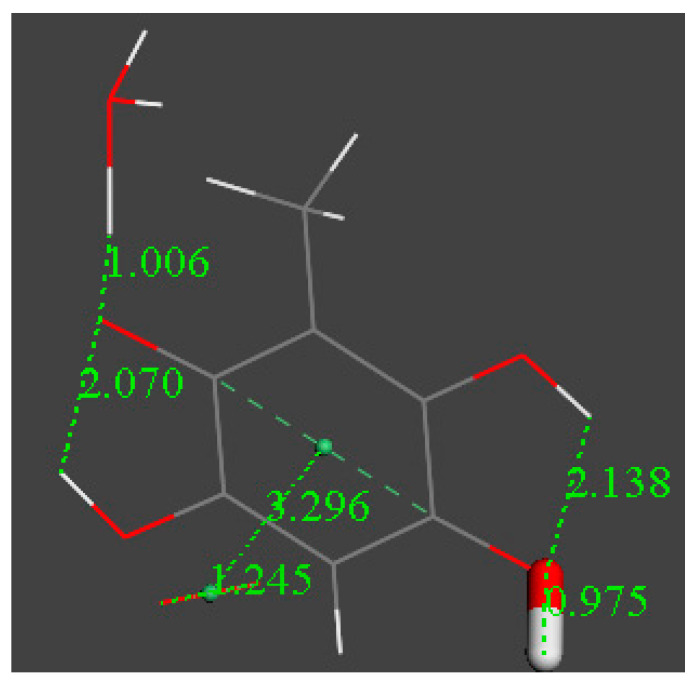
An additional H_3_O^+^ is reacted at the remaining O(carbonyl) of [embelin-model-H]^−^, and the proton is also captured. ΔG = −38.3 kcal/mol.

**Figure 10:**
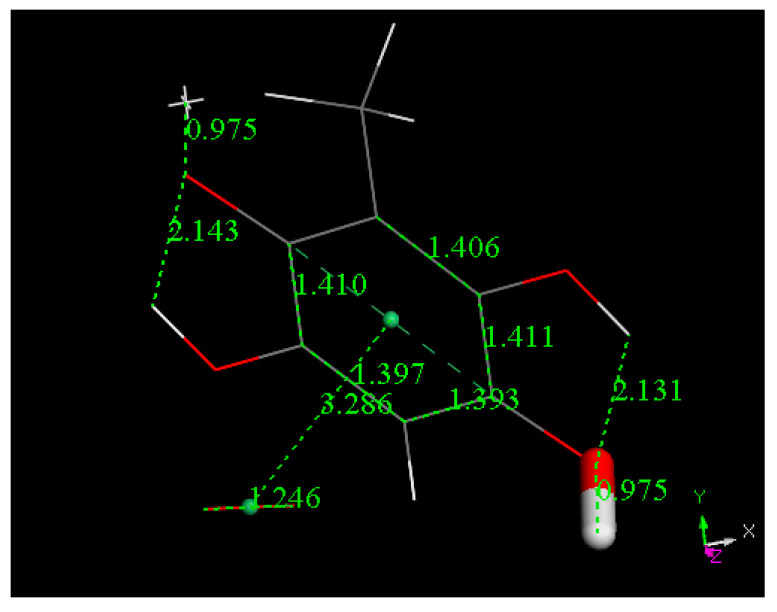
H_2_O was eliminated from the system shown in Figure 9 and a geometry minimization performed.

**Figure 11:**
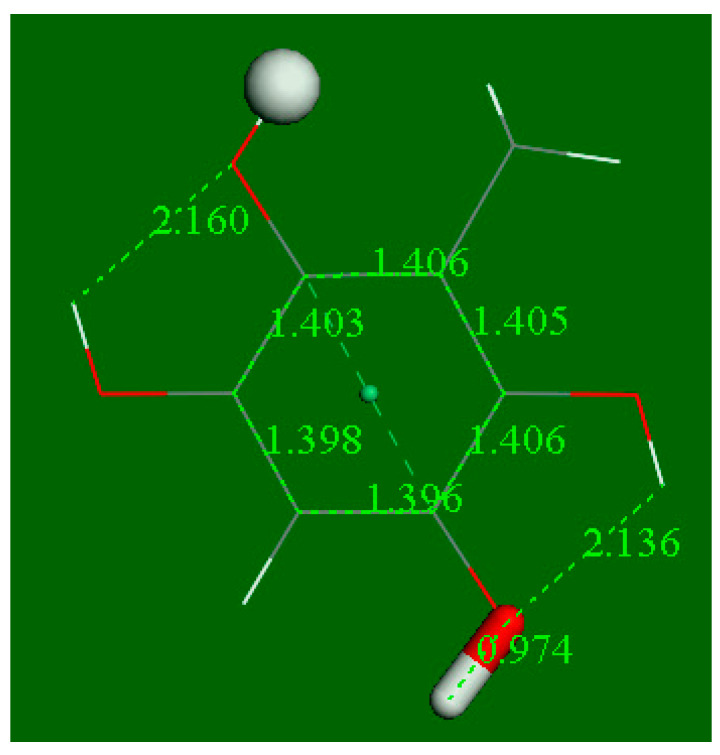
After eliminating H_2_O from Figure 9, the final product is a double catechol hydroquinone. ΔG = −15.0 kcal/mol, no barrier.

To better discern embelin’s mode of action, its approach towards superoxide was further analyzed. Earlier studies demonstrated [8] that embelin accepts an electron from superoxide after a π–π interaction and becomes a radical species, [embelin]^−•^. A potential subsequent step, where a proton interacts with [embelin]^−•^, was analyzed using a model where the embelin C_10_ alkyl tail was replaced by a methyl group (named embelin-model). Indeed, after geometry minimization, a proton is thermodynamically accepted with the formation of a neutral radical, [embelin-model-H]^•^, and the geometry minimization proceeded without an energy barrier, as shown in Figure 7. In our next calculation, another superoxide was brought towards the scavenger species, and its electron was captured, as shown in Figure 8. We expected this species to have another potential proton addition, and indeed, this also resulted to be thermodynamically feasible, without an energy barrier, as shown in Figure 9. Since we used a hydronium cation for this calculated reaction, the released water molecule was eliminated, as shown in Figure 10, and the final minimized product molecule, after excluding the formed O_2_ molecule, is presented in Figure 11.

The net reaction is therefore (embelin model = 2,5-diol-benzoquinone, 3-methyl);

2 O_2_^−^(rad) + 2 H_3_O^+^ + embelin-model → 1,2,4,5-benzenetetrol, 3-methyl + 2 O_2_ + 2 H_2_O

or

2 O_2_^−^(rad) + 2 H^+^ + embelin-model → 1,2,4,5-benzenetetrol, 3-methyl + 2 O_2_

Our calculations use a model of embelin, and the resulting calculated derivative (1,2,4,5-benzenetetrol, 3-methyl) has been previously described [24,25], and so the existence of the corresponding embelin derivative is feasible. 

**Figure 12:**
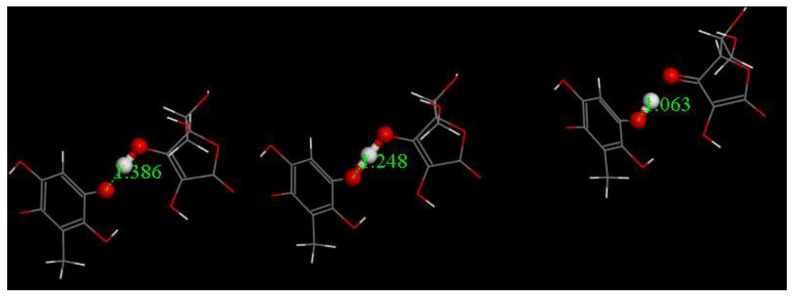
Reagent, Transition State and Product for semiquinone [embelin] ^•−^ capturing H^+^ from vitamin C to form embelin-H. ΔG = −0.8kcal/mol, E_Barrier_ = 0.2 kcal/mol.

**Figure 13:**
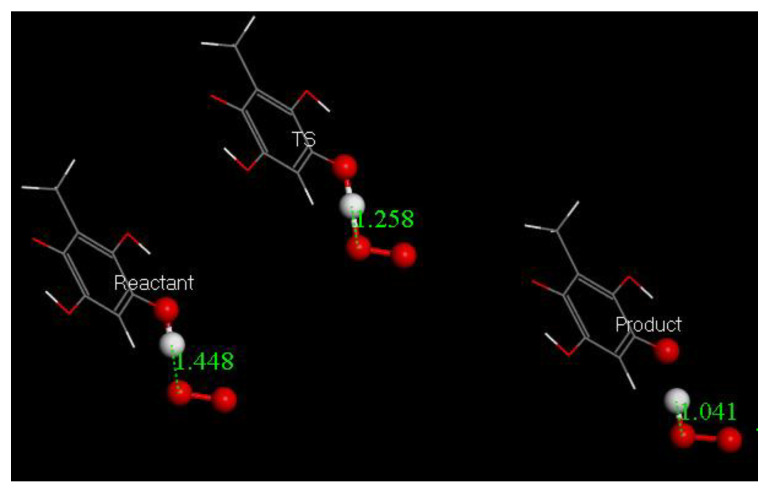
Reactant, Transition state and Product of embelin derivative (2,4,5-benzenetriol, 3-methyl). The scavenger donates H to O_2_^•−^ forming an aromatic radical and releasing HO_2_^−^, which in turn will become H_2_O_2_, after capturing a proton. ΔG = −1.2 kcal/mol and E_Barrier_ = 0.3 kcal/mol. Additional info provided in Figure S2.

Embelin capture of an electron from superoxide, for instance, that depicted in the Figure 7 calculation, uses a hydronium (proton), which may be ascribed to a strong acidic environment. Such an environment probably does not exist in the mitochondria and so we tested the same reaction for a more representative weaker acid—vitamin C (pKa = 4.17), and this process turned out to be thermodynamically feasible, as shown in Figure 12. The calculated product (2,4,5-benzenetriol, 3-methyl) has a catechol moiety that may easily perform additional superoxide scavenging, and which can explain the strong activity of embelin in Figure 3. This reaction is shown in Figure 13.

Therefore, according to these calculations, embelin is able to scavenge superoxide in a versatile manner, initially through a π–π capture of the superoxide electron, to release O_2_ (for instance, Figure 8), and later, after capturing protons, by a σ interaction following the typical release of H and forming HO_2_^−^, shown in Figure 9. 

This dual capability of embelin for superoxide scavenging agrees with the experimental RRDE result. Embelin completely eliminates superoxide, and is, in fact, stronger than BHT, as shown by their slopes for less than 50 × 10^−5^ M of antioxidant, shown in Figure 3. The DMBQ scavenging of superoxide is weaker than embelin and the former can be only associated to π–π reactivity, but it is still stronger than BHT. In conclusion, embelin may involve both scavenging actions initially through π–π reactivity, followed by proton capture from an acidic environment in the cell, and this is consistent with the better performance of embelin in the RRDE experiment compared with DMBQ. The yield of this sequence of reactions is probably limited in our RRDE experiment, since the availability of protons (hydronium cations in our DFT study) is scarce, as the solvent is preferentially anhydrous. However, in a biological environment, proton availability is more feasible, and so a strong antioxidant biological capability of embelin may be expected.

### 3.3. Modeling Cell Membrane Interaction of Embelin

**Figure 14:**
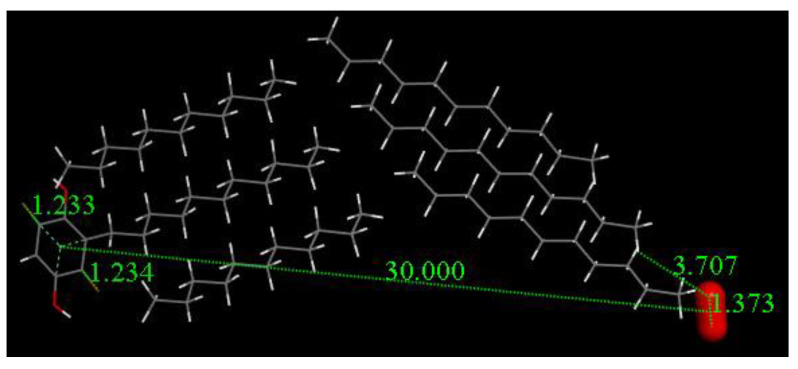
Embelin model for insertion in cell membrane. The superoxide molecule (d(O-O) = 1.373 Å) is posed to run a geometry minimization.

Taking advantage of the crystal structure data of embelin, we build a model for cell membrane interaction. We consider the packing of the long lipophilic alkyl tail of embelin as a model for embelin’s ability to insert itself in the membrane cell. Figure 14 shows six molecules of embelin as found in its crystalline cell [8]; five of these molecules have the quinone moiety removed, so only the alkyl tail remains. This system allows us to simulate a model for the cell membrane double layer by considering the three molecules on the right of Figure 14 as belonging to the inner cell part, and the two molecules on the left to the outer cell. We ascribe the single molecule containing the quinone moiety to represent an embelin molecule inserted within the double layer. We place a superoxide radical “inside” the cell, at van der Waals separation from one of the three units, to mimic what happens in the mitochondria when such superoxide gets out of cell control, ready to leak through the membrane. 

**Figure 15:**
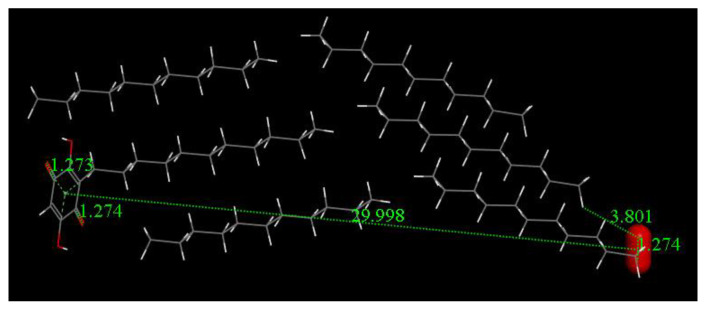
Distances on embelin ring moiety are the carbonyl C-O bonds, 1.274 Å and 1.273 Å. These are lengthened from the values before reaction, 1.224 Å and 1.233 Å, as seen in Figure 14.

Figure 15 shows the result of a geometry optimization for the system depicted in Figure 14. The geometry of the quinone moiety appears to be affected by the presence of the superoxide, consistent with our previous study on the π–π interaction, e.g., lengthening of quinone C = C double bonds and shortening of C-C bonds. This sort of quinone ring aromatization is associated with a change in the superoxide structure. Thus, in the inner part of the cell, the O-O bond length is shortened (1.274 Å from superoxide 1.373 Å), consistent with data shown already for the π–π approach [8]. Thus, the quinone captures the electron from superoxide, even at a distance far from van der Waals separation. These results suggest a potential way for embelin to circumvent superoxide leaking due to electron capture from superoxide. 

**Figure 16:**
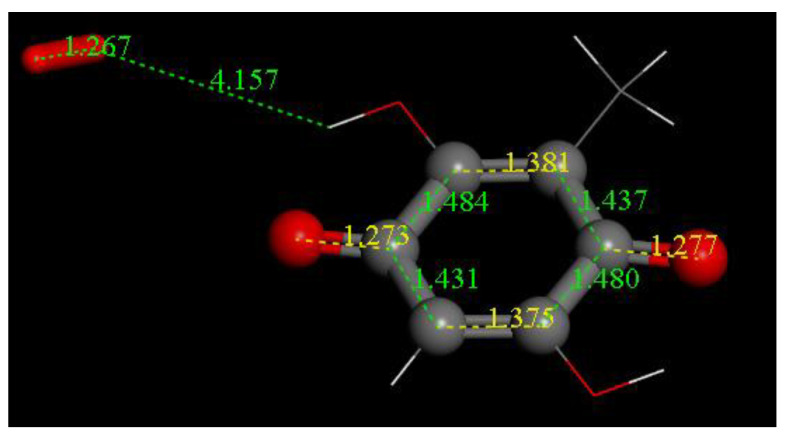
After superoxide radical (O-O bond distance 1.373 Å) was initially placed 4 Å apart from one embelin hydroxyl, the system was geometry minimized and the superoxide released its electron to the quinone ring, that is, performing a σ capture.

However, so far, we identified embelin’s initial reaction with superoxide through a π–π interaction, which differs from what is shown in Figure 15. Therefore, a calculation with reagents at a shorter range was performed, shown in Figure 16, which confirmed a σ scavenging mode of superoxide.

### 3.4. Antioxidant and Biological Activity of Embelin in Cells in Culture 

**Figure 17:**
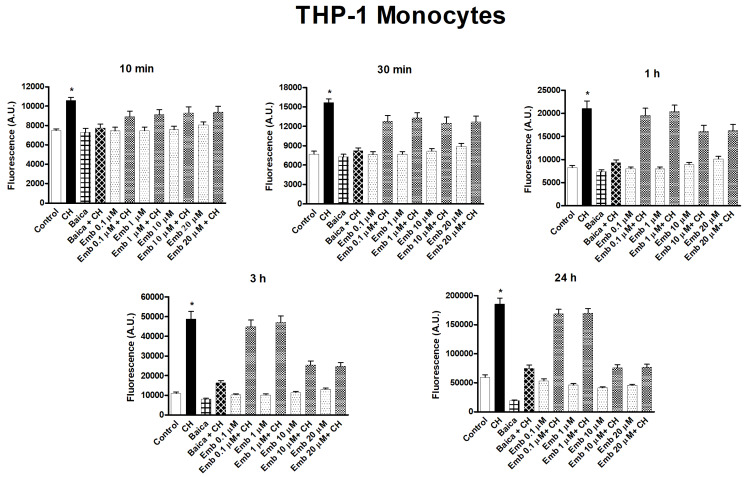
Time course of embelin capability to protect toward oxidative stress given by Cumene hydroperoxide in THP-1 monocytes. The concentration of cumene hydroperoxide (CH) was 200 µM for both cell lines. Before the addition of cumene hydroperoxide, cells were pre-incubated with embelin (0.1 μM–20 μM) at 37 °C for 10 min, as reported in Section 2.6. Baicalein 10 μM was used for both cell lines, as reference compound, showing a very good antioxidant activity [18]. The results are mean ± SD of at least three different experiments carried out in quintuplicate on 96 wells plates. A period of 10 min, 30 min, *p < 0.01, at least, vs. all but embelin + CH; 1 h, 3 h, 24 h, *p < 0.05 at least, vs. all but embelin 0.1 μM + CH and embelin 1 μM + CH.

**Figure 18:**
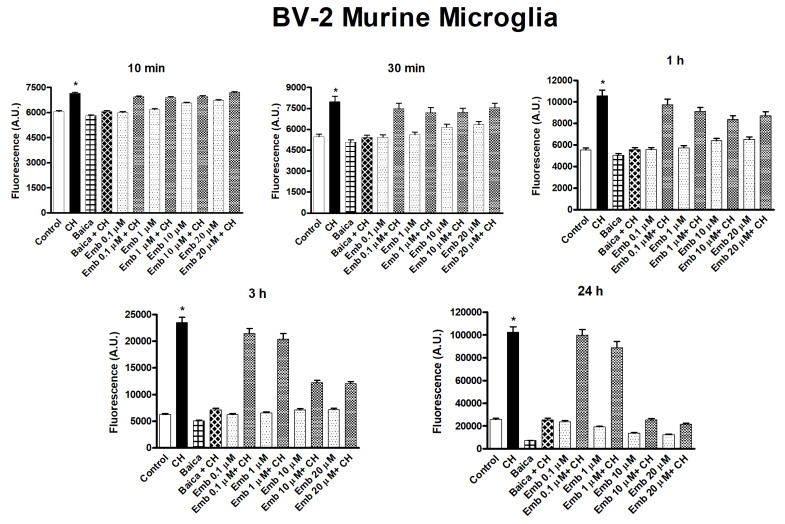
Time course of embelin capability to protect toward oxidative stress given by Cumene hydroperoxide in BV-2 murine microglia cells. The concentration of cumene hydroperoxide (CH) was 200 µM for both cell lines. Before the addition of cumene hydroperoxide, cells were pre-incubated with embelin (0.1 μM–20 μM) at 37 °C for 10 min, as reported in Section 2.6. Baicalein 10 μM was used for both cell lines, as reference compound, showing a very good antioxidant activity [18]. The results are mean ± SD of at least three different experiments carried out in quintuplicate on 96 wells plates. A period of 10 min, 30 min, *p < 0.01, at least, vs. all but embelin + CH; 1 h, 3 h, 24 h, *p < 0.05 at least, vs. all but embelin 0.1 μM + CH and embelin 1 μM + CH.

**Figure 19:**
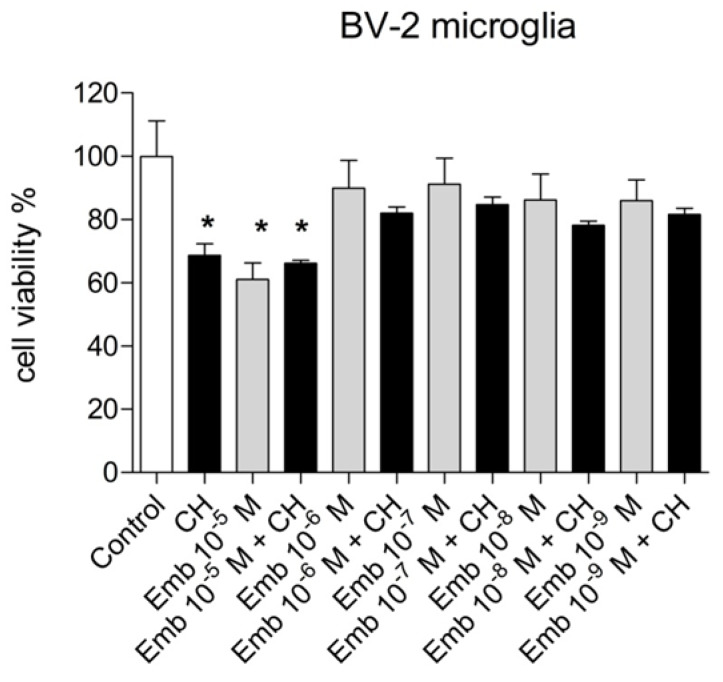
Cytotoxicity/proliferation (the MTT) assay carried out with embelin in the range 10^−6^–10^−9^ M in BV-2 murine microglia. The concentration of cumene hydroperoxide (CH) was 200 µM throughout the experiments. Results are the mean ± SD of two different experiments carried out in quintuplicate. *p < 0.01 at least vs. Control and embelin (10^−6^–10^−9^ M) either alone or with CH.

**Figure 20:**
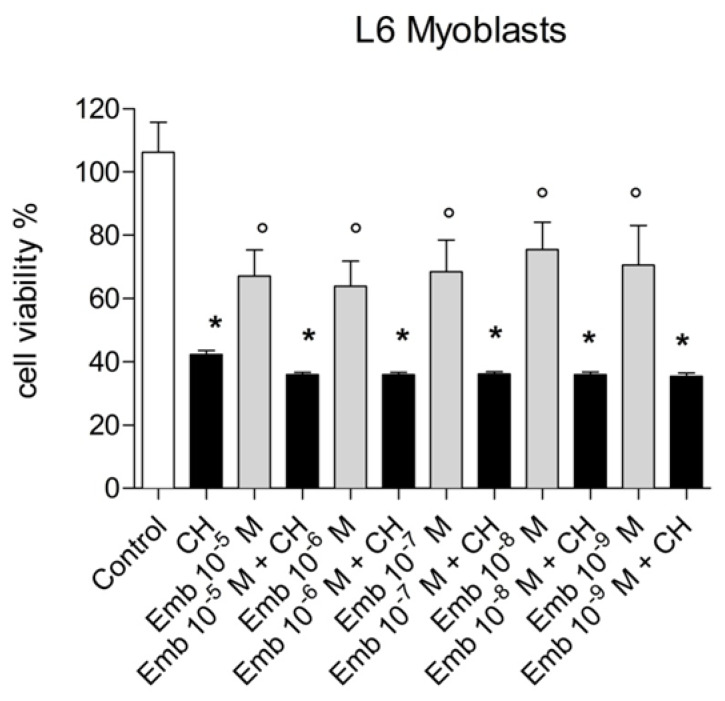
Cytotoxicity/proliferation (the MTT) assay carried out with embelin in the range 10^−6^–10^−9^ M in L6 myoblasts. The concentration of cumene hydroperoxide (CH) was 27.5µM throughout the experiments. Results are the mean ± SD of two different experiments carried out in quintuplicate. *p <0.001 vs. all, °*p* < 0.01 vs. control.

We evaluated the capability of embelin to protect the cells from oxidative stress given by cumene hydroperoxide in THP-1 human leukemic monocytes and BV-2 mice microglia cells in a wide concentration range 0.1 µM–20 µM and for different times (10 min–24 h) (Figure 17 and Figure 18). Embelin alone, in the short time range (up to 30 min), was not able to protect the cells from oxidative stress given by cumene hydroperoxide (200 µM), but at a longer time, the highest concentrations were able to protect the cells from oxidative stress. In particular, at 24 h, the value of ROS produced after cumene hydroperoxide addition in the presence of embelin reached the basal control level for the highest concentrations (Figure 17 and Figure 18). This indicates that embelin probably acts in the classical mode of activation of antioxidant defense systems through the modulation of gene transcription as reported [26,27]. The results obtained with embelin were similar to those of baicalein, known to be an excellent antioxidant compound. Similar data were obtained with THP-1 monocytes. We also evaluated the possible cytotoxicity of embelin for both cell lines by MTT assay (Figure 19 and Figure 20). Embelin at low concentrations showed protection in BV-2 murine microglia toward the stress induced by cumene hydroperoxide. In the L6 myoblasts, embelin alone showed some toxicity (30%) that was similar to that of cumene at 27.5 µM alone. In the presence of cumene at any concentration, embelin did not protect the L6 myoblasts from oxidative stress, but a sum of the two toxicities appeared when the two compounds (embelin and cumene) were given together. 

**Figure 21:**
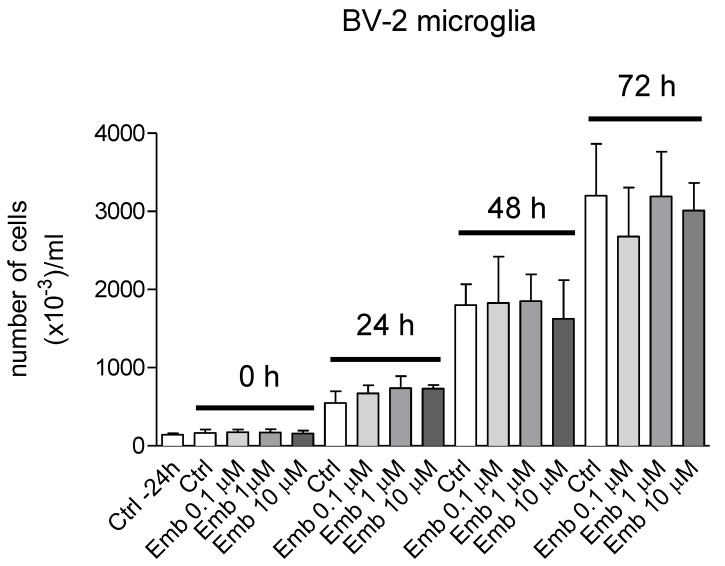
Proliferation curves in the presence of embelin (0.1–10 μM) of BV-2 microglia. BV-2 microglia cells were seeded in 60 × 15 mm Petri dishes in 4 mL of the respective medium at appropriate density to reach confluence after 96 h. Cells were stimulated with embelin 24 h from seeding and then counted every 24 h up to confluence. Results are the mean ± SD of 3 different experiments carried out in triplicate. None of the reported results showed significance within the same group.

**Figure 22:**
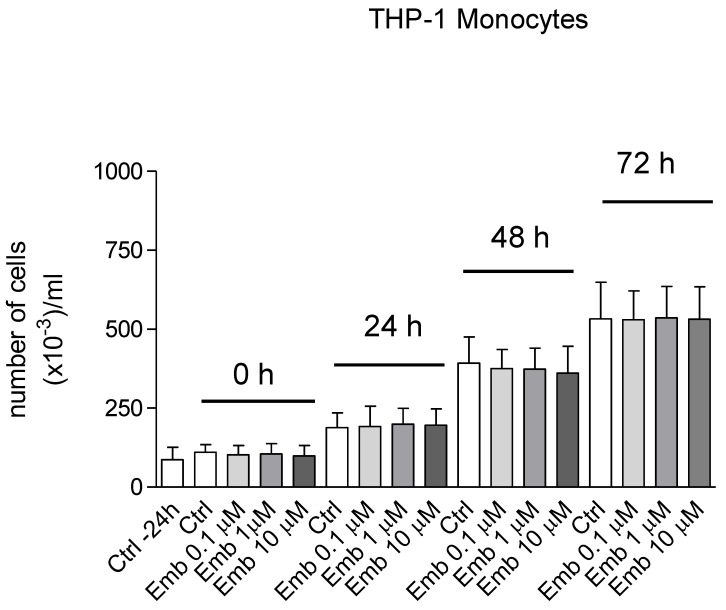
Proliferation curves in the presence of embelin (0.1–10 μM) of THP-1 human leukemic monocytes. The THP-1 monocytes growing in suspension were seeded in 24 wells plates with 1 mL complete medium. Cells were stimulated with embelin 24 h from seeding and then counted every 24 h up to optimal density (96 h). Results are the mean ± SD of 3 different experiments carried out in triplicate. None of the reported results showed significance within the same group.

We also carried out the proliferation curve both with BV-2 and THP-1 monocytes in the presence and absence of embelin and at the concentrations employed. The cell proliferation was not affected by embelin in both cell lines (Figure 21 and Figure 22).

## 4. Discussion 

In this work, we studied the embelin scavenging of superoxide with our recently developed RRDE method and found that embelin is a much stronger antioxidant than the commercially used BHT. DFT results confirm that embelin is able to perform superoxide scavenging through the reduction of its quinone ring while producing molecular oxygen. Indeed, a recent study by our group [22] shows that another quinone, emodin, acts through the same reduction pathway when interacting with superoxide. A marked difference is observed when comparing embelin with its bis-methoxylated derivative, DMBQ: embelin is a much stronger sequestering agent than DMBQ. This suggests that the molecular structure of embelin allows for additional reactivity with superoxide, besides the π–π interaction. Further reactivity between embelin and an additional superoxide radical, mediated by a proton, is predicted by DFT and the suggested mechanism supports the restoration of embelin, consistent with its superior performance as a scavenger. That embelin is a very strong antioxidant, is indeed shown by the complete depletion of superoxide in the RRDE voltaic cell. 

We show here that embelin protects the cells from oxidative stress generated by cumene hydroperoxide in THP-1 human leukemic monocytes and BV-2 mice microglia cells in a wide concentration range 0.1–20 µM (Figure 17). The unusual aspect for a small molecule endowed of antioxidant activity is that embelin alone, in the short time range (up to 30 min), did not protect the cells from oxidative stress given by cumene hydroperoxide (200 µM), but at a longer time the highest concentrations were able to protect the cells. In particular, at 24 h, the value of ROS produced by cumene in the presence of embelin returned to the basal control level for the highest concentrations, as shown in Figure 17. This indicates that embelin probably acts as an antioxidant through different processes, for example, by the modulation of gene transcription of the antioxidant defense system, as already reported [26,27] or via other mechanisms. In our experiments, we compared the effect of embelin with baicalein, a very good antioxidant with three OH groups [18,28,29], given that our earlier studies had shown that baicalein was the most effective of the different flavone compounds studied, including the flavonol quercetin [18]. A comparison of embelin antioxidant activity with other small plant compounds isolated from the fruits of *Ardisia colorata* (Myrsinaceae), a Thai medicinal plant, showed it was superior in DPPH radical scavenging activity [30]. A more recent paper describes embelin superiority over a variety of phytoceutical compounds in helping towards the normalization of pancreatic tissue [31] since it favors the apoptosis with respect to necrosis. 

Since embelin biological antioxidant action needs a longer time with respect to other small molecules scavengers analyzed by our group [18,28], we suspected that the long tail of embelin could slow molecular mobility in the biological environment and be responsible for this delay. Taking advantage of the X-ray crystal structure of embelin [8], we built a model to mimic the cell membrane insertion of this scavenger. Our DFT calculation shows that the scavenging of superoxide is feasible in this model, with embelin acting at a long range, that is, intercepting superoxide and capturing its electron. Thus, embelin is inferred to act at a long distance from superoxide through the membrane, hence avoiding superoxide leakage from the cell. Since this process transforms superoxide into O_2_, its reaction with embelin results in a safe outcome. This computational result is consistent with the biological study, showing that the inhibition of cumene hydroperoxide-induced peroxidation of human leukemic cells by embelin does not occur *immediately*. That is, a certain interval is needed for embelin biological scavenging in our cell experiment, and we suggest that this time delay can be associated to the accommodation of embelin in the cell membrane. An explanation of the delay in antioxidant activity reported in our experiments could also be due to other physiological processes. In fact, embelin has been reported to act as an antioxidant by stimulating the synthesis of antioxidant defense systems such as SOD, catalase, GSH in a high fat diet and streptozotocin-induced diabetes in Wistar rats [26,27].

Our results, obtained with embelin in cells in culture, are similar to those of baicalein, known to be an excellent antioxidant compound [18,28,29]. We also evaluated the capability of embelin microglia [32] to be cytotoxic for the cells or modulate proliferation by MTT assay and proliferation curves. Embelin, by itself, was not toxic, and was able to protect BV-2 murine microglia. At the same time, embelin did not affect cell proliferation, Figure 21 and Figure 22.

In conclusion, embelin shows good protective capability in the MTT assay towards cumene hydroperoxide-stress induced in BV-2 cells. It is important to recall that the MTT assay evaluates the functional activity of the mitochondrial succinate dehydrogenase. Therefore, the result depends on mitochondria respiration and reported data confirm the protective effects of embelin versus oxidative stress given to microglia cells. The mitochondrial respiratory rate or oxygen consumption rate (OCR) was increased by embelin (1 – 10 μM) at 16 – 24 h in N27 dopaminergic cells. The effect is due to increased levels of pAMPK, SIRT1, and PGC1α, leading to increased mitochondrial biogenesis [12]. Only one paper has been reported, to our knowledge, describing the effects of embelin in microglia [32]. Our data are among the first reported in normal microglia, the immune system of the central nervous system, and since embelin and its derivatives can easily cross the blood–brain barrier, embelin could represent a feasible pharmacological tool toward several types of disorders, such as neurodegenerative diseases, where the mitochondria functionality may play a pivotal role [2,3,12,33,34]. 

## Figures and Tables

**Table 1:** Slopes of collection efficiency from Figure 3.

Compound	Toluene	BHT	DMBQ	Embelin
Slope	0	−1.6 × 10^3^	−1.6 × 10^4^	−6.0 × 10^4^

## Supplementary Materials

The following are available online at https://www.mdpi.com/2076-3921/9/5/382/s1, Figure S1: Toluene RRDE voltammogram shows no scavenging of superoxide, Figure S2: Transition State search for superoxide scavenged by 1-oxo,2,4,5-benzenetriol, 3- methyl). ΔG = −1.2 kcal/mol and EBarrier = 0.3 kcal/mol.
