# Supplementary material for: Antioxidant Properties of Embelin in Cell Culture. Electrochemistry and Theoretical Mechanism of Scavenging. Potential Scavenging of Superoxide Radical through the Cell Membrane
Source: Antioxidants (Basel). 2020 May 5;9(5):382. doi: 10.3390/antiox9050382 (PMC7278612; doi:10.3390/antiox9050382)
Supplement: Supplementary file 1 [file antioxidants-09-00382-s001.pdf]

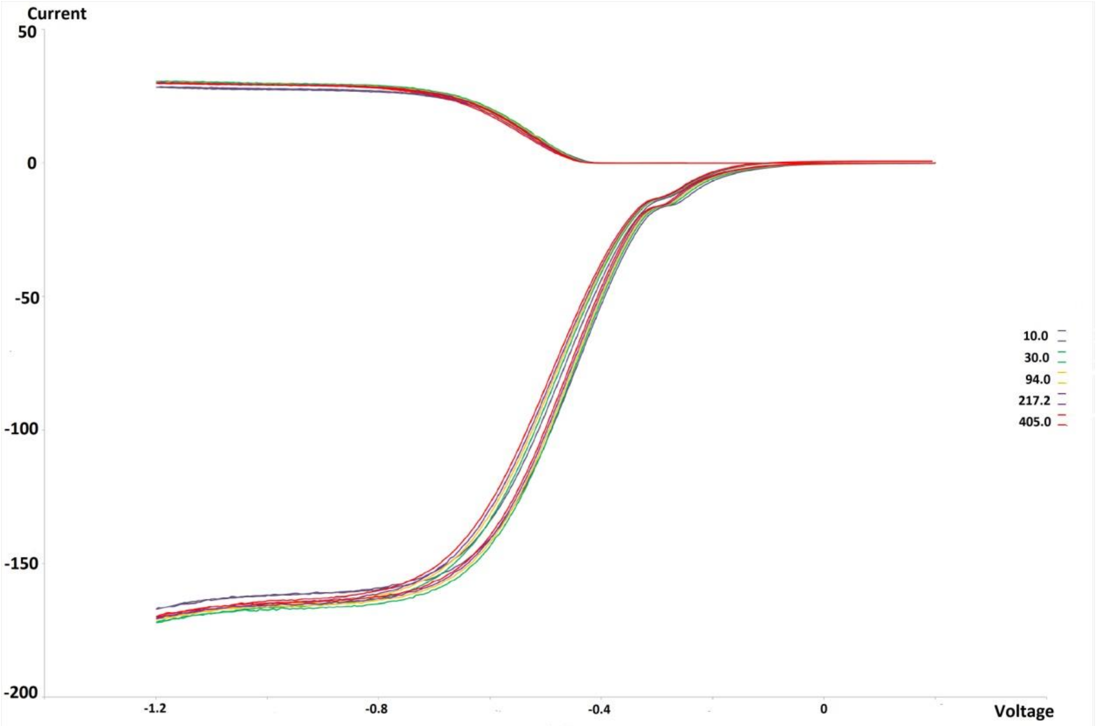

Figure S1. Toluene RRDE voltammogram shows no scavenging of superoxide.

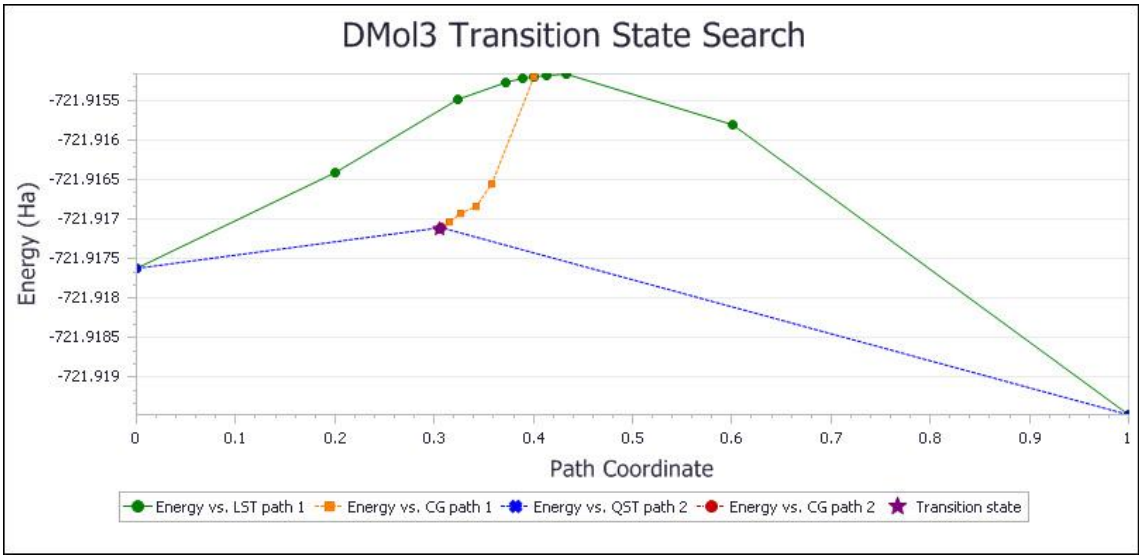

Figure S2. Transition State search for superoxide scavenged by 1-oxo,2,4,5-benzenetriol, 3- methyl).

ΔG = -1.2 kcal/mol and $E_{Barrier}$ = 0.3 kcal/mol.
